# DNMT1-mediated PPARα methylation aggravates damage of retinal tissues in diabetic retinopathy mice

**DOI:** 10.1186/s40659-021-00347-1

**Published:** 2021-08-06

**Authors:** Ying Zhu, Xinru Wang, Xiaoyun Zhou, Lexi Ding, Dan Liu, Huizhuo Xu

**Affiliations:** 1 Eye Center of Xiangya Hospital, Central South University, No. 87 Xiangya Road, Hunan 410008 Changsha, China; 2 Department of Ophthalmology, The First Hospital of Changsha, 311 Yingpan Road, Hunan Changsha, China

**Keywords:** DNMT1, PPARα, DNA methylation, Apoptosis, Diabetic retinopathy

## Abstract

**Background:**

Peroxisome proliferator-activated receptor alpha (PPARα) is associated with diabetic retinopathy (DR), and the underlying mechanism is still unclear. Aim of this work was to investigate the mechanism of PPARα in DR.

**Methods:**

Human retinal capillary pericytes (HRCPs) were treated with high glucose (HG) to induce DR cell model. DR mouse model was established by streptozotocin injection, and then received 5-Aza-2-deoxycytidine (DAC; DNA methyltransferase inhibitor) treatment. Hematoxylin–eosin staining was performed to assess retinal tissue damage. PPARα methylation was examined by Methylation-Specific PCR. Flow cytometry and DCFH-DA fluorescent probe was used to estimate apoptosis and reactive oxygen species (ROS). The interaction between DNA methyltransferase-1 (DNMT1) and PPARα promoter was examined by Chromatin Immunoprecipitation. Quantitative real-time PCR and western blot were performed to assess gene and protein expression.

**Results:**

HG treatment enhanced the methylation levels of PPARα, and repressed PPARα expression in HRCPs. The levels of apoptotic cells and ROS were significantly increased in HRCPs in the presence of HG. Moreover, DNMT1 was highly expressed in HG-treated HRCPs, and DNMT1 interacted with PPARα promoter. PPARα overexpression suppressed apoptosis and ROS levels of HRCPs, which was rescued by DNMT1 up-regulation. In DR mice, DAC treatment inhibited PPARα methylation and reduced damage of retinal tissues.

**Conclusion:**

DNMT1-mediated PPARα methylation promotes apoptosis and ROS levels of HRCPs and aggravates damage of retinal tissues in DR mice. Thus, this study may highlight novel insights into DR pathogenesis.

**Supplementary Information:**

The online version contains supplementary material available at 10.1186/s40659-021-00347-1.

## Background

Diabetic retinopathy (DR) is one of the major causes of blindness, and the most common retinal vascular disease [1]. In recent years, the incidence of diabetes and DR has increased year by year, which is a serious threat to human health [2]. In China, the prevalence of DR in diabetic patients is 20–40% [3]. By 2040, there will be nearly 600 million people with diabetes in the world, and one-third of the population will have the performance of DR [4, 5]. The changes in blood composition of diabetic patients cause dysfunction of vascular endothelial cells, which damages the blood-retinal barrier [6]. The blood-retinal barrier is a restrictive permeable structure that exists between the blood and the retina, and it plays a vital role in ensuring the stability of the retina environment and normal visual function [7]. Increased retinal vascular permeability caused by blood-retinal barrier damage is an important pathological feature in the early stage of DR [8]. In clinical, the onset of DR is relatively insidious, and there is generally no eye symptom in the early stage. Thus, a good time for DR treatment is delayed. At present, the pathogenesis and treatment of DR have always been the difficulties and hot spots in ophthalmology clinical and basic research [9].

The blood glucose levels of diabetic patients have a great influence on diabetic complications. Random clinical trials have shown that intensive control of blood glucose by injecting insulin to ensure that blood glucose levels are in the non-diabetic range (3.9–6.1 mM), which effectively reduces the incidence of myocardial infarctions, stroke, and cardiovascular disease death [10, 11]. However, if a diabetic patient with hyperglycemia for a long time, even if the levels of blood glucose are controlled in the later stage, diabetes-related complications are still prone to occur. This phenomenon is called “metabolic memory” [12–14]. The existence of “metabolic memory” has become a major obstacle to diabetic complication treatment, but its mechanism has not been fully elucidated. Many scholars have found that “metabolic memory” is associated with epigenetics [15–17]. Epigenetics reflects the heritable changes in gene expression caused by non-DNA sequence changes, and it embodies the two important concepts of “environment” and “genetic”. Miao et al. have clarified the correlation between HbA1c and H3K9ac modifications in the peripheral blood lymphocytes and monocytes of type 1 diabetes mellitus patients [12]. High glucose induces persistent hypomethylation of TXNIP in THP1 cells [18]. Thus, epigenetics is expected to become an important breakthrough in revealing the phenomenon of “metabolic memory”, thereby providing new insights for diabetes complication treatment.

As the main content of epigenetics, DNA methylation modification participates in many pathophysiological processes such as embryonic development, stem cell differentiation, genome imprinting, tumorigenesis, inflammation and aging [19]. DNA methyltransferase-1 (DNMT1) is a key enzyme to maintain DNA methylation [20]. DNMT1-mediated MEG3 methylation takes part in the progression of DR [21]. Mishra and Kowluru have found that the global DNA methylation levels are relatively higher in patients with DR [22]. Peroxisome proliferator-activated receptors (PPARs) are a group of transcription factors that belong to the nuclear hormone receptor family. The genes regulated by PPARs are involved in lipid metabolism and insulin resistance [23]. PPARα participates in the regulation of oxidative stress, inflammation and vascular function [24–26]. Previously, our data has confirmed that fenofibrate inhibits apoptosis of human retinal capillary pericytes (HRCPs) in the early stage of DR by activating PPARα [27]. In addition, PPARα is significantly down-regulated in the retina of diabetic patients and high glucose (HG)-treated HRCPs. PPARα inhibition aggravates microangiopathy and inflammation in streptozotocin (STZ)-induced DR mouse model [28]. Moreover, dietary protein restriction of pregnant rats reduces the DNA methylation levels of PPARα in hepatocytes of offspring [29]. However, a high-fat diet increases the DNA methylation levels of PPARα in mice and oocytes of their offspring [30]. Thus, PPARα methylation may participate in the development of DR. The purpose of this article is to investigate the biological role of PPARα methylation in regulating apoptosis of HG-induced HRCPs. We further verified that whether inhibition of DNA methylation of PPARα can reduce damage of retinal tissues in DR mice.

## Materials and methods

### Animals

C57BL/6 J male mice with 6–8 weeks old (weighting 20–24 g) were purchased from Beijing Vital River Laboratory Animal Technology Co., Ltd. (China). C57BL/6 J mice were housed under specified pathogen free conditions. Animal experiments were conducted in accordance with the Association for Research in Vision and Ophthalmology Statement for the Use of Animals in Ophthalmic and Vision Research, and authorized by the Ethics Committee of Xiangya Hospital, Central South University (No. 201904609; Changsha, China).

C57BL/6 J male mice were divided to three groups (n = 8): (1) Control group: normal mice were served as control; (2) DR group: DR mouse model was induced by intraperitoneal injection of STZ (50 mg/kg). After 72 h of intraperitoneal injection, the glucose level of tail vein blood in the mice was examined using automated Accu-Chek glucometer (Roche Diagnostics, Basel, Switzerland) every 2 weeks. The glucose level of mice more than 250 mg/dL was deemed as DR mice, and the DR mice were raised for 10 weeks; and (3) DAC group: the DR mice were intraperitoneally injected with 5-Aza-2-deoxycytidine (DAC; 1 mg/kg·d) every day for the last 4 weeks.

After modeling, the mice were euthanized by cervical dislocation. The retinal tissues of mice were rapidly excised and snap-frozen in the liquid nitrogen. The retinal tissues were stored at − 80 °C for further use.

### Cell culture

HRCPs (Cambrex Biosciences, Walkersville, MD, USA) were cultured in DMEM (Solarbio, Beijing, China) at 37°C and 5% CO_2_. DMEM contained 10% fetal bovine serum (FBS) and 1% penicillin/streptomycin (Solarbio). HRCPs were incubated with 5 mM glucose (normal glucose, NG), 30 mM glucose (HG) or 5 mM glucose + 25 mM mannose (Man) with or without 10 mM DAC for 4 d.

### The methylation levels of PPARα

The methylation levels of PPARα in HRCPs were detected by performing Methylation-Specific PCR (MSP). The genome DNA was extracted from HRCPs using Universal Genomic DNA Extraction Kit (Solarbio). The methylation levels of DNA samples were examined using CpGenome Universal DNA Modification (Merck Millipore, Billerica, MA, USA) following the instruction of manufacturer. The primers set specific to the methylated DNA and un-methylated DNA were purchased from GeneChem (Shanghai, China). The primer sequences (5′–3′) were shown as follows: PPARα (Methylation): forward: AGAGTAGTAGAGTCGGGTTTATCGA; reverse: GAAACGAAACTAAATTCGAAACG. PPARα (Unmethylation): forward: AGAGTAGTAGAGTTGGGTTTATTGA; reverse: AAAACAAAACTAAATTCAAAACAAA.

### Cell transfection

The full length of PPARα generated from genome DNA of HRCPs was subcloned into the vector pCEP4, generating the vector pCEP4-PPARα (GeneChem). The empty pCEP4-NC vector served as control (vector1). The vector pcDNA3.1-DNMT1 overexpressed DNMT1, and the corresponding empty vector pcDNA3.1-NC (vector2) were constructed by GeneChem. Small interference RNA (siRNA) specifically targeting PPARα (si-PPARα-1 and si-PPARα-2) or DNMT1 (si-DNMT1-1 and si-DNMT1-2), and the corresponding NC (siRNA) were purchased from GeneChem. HRCPs (100 μL; 1–5 × 10^4^ cells) were transfected with 75 ng siRNA/miRNA using 3 μL HiPerFect Transfection Reagent (Qiagen, Hilden, Germany), or transfected with 2 μg vector using 2 μL Lipofectamine 2000 Reagent (Invitrogen, Carlsbad, CA, USA) as the protocol described. After 48 h of transfection, the modified HRCPs were collected and stored at − 20 °C for further use.

### Gene expression

Quantitative real-time PCR (qRT-PCR) was used to measure the gene expression in HRCPs and mouse retinal tissues. TRIzol reagent (Invitrogen) was used to extract total RNA from HRCPs and mouse retinal tissues. RNA integrity was examined by 1.5% agarose gel electrophoresis. RNA was reverse transcribed using PrimeScript™ RT reagent Kit (Takara, Tokyo, Japan) to synthesis complementary DNA. QRT-PCR was performed applying TB Green^®^ Premix Ex Taq™ II (Tli RNaseH Plus) (Takara) on an ABI Prism 7000 system (Thermo Fisher Scientific, Waltham, MA, USA). The relative expression of genes was analyzed using 2^−∆∆CT^ method.

### Protein expression

Protein expression in HRCPs and mouse retinal tissues was examined by performing western blot (WB) assay. Total protein was extracted from HRCPs and mouse retinal tissues using Total Protein Extraction Kit (Solarbio) as the introduction described. BCA Protein Assay Kit (Solarbio) was used to examine the concentration of proteins. Protein samples were separated by 10% SDS-PAGE electrophoresis. Subsequently, the separated proteins were transferred onto polyvinylidene fluoride membranes (Merck Millipore). After blocked with 5% skim milk, the membranes were incubated with the primary antibodies, PPARα, DNMT1, DNMT3A or DNMT3B (1:1000; Proteintech, Wuhan, China), at 4 °C for 12 h. Next, the membranes were incubated with the horseradish peroxidase-conjugated secondary antibody (1:5000; Proteintech). β-actin antibody (1:5000; Proteintech) was used as a reference protein for normalization. The data were analyzed by Image J software.

### Cell apoptosis

Apoptosis of HRCPs was examined applying Annexin V-FITC/PI Apoptosis Detection Kit (YEASEN, Shanghai, China). HRCPs were collected and washed with phosphate buffer saline (PBS) for several times. HRCPs were resuspended in 100 μL 1× Binding Buffer. The cell suspension was incubated with 5 μL Annexin V-FITC and 10 μL PI staining solution at darkness for 15 min. Subsequently, the cell suspension was mixed with 400 μL of 1× Binding Buffer and put on ice. Cell apoptosis was determined by flow cytometry (BD Biosciences, San Jose, CA, USA) in an hour.

### Detection of intracellular reactive oxygen species (ROS)

The levels of ROS in HRCPs were examined using Reactive Oxygen Species Assay Kit (Solarbio) as the protocol of the manufacturer. HRCPs were seeded in 24-well plates at a concentration of 2 × 10^4^ cells/well and cultured for 24 h. HRCPs were incubated with 1 mL DCFH-DA (10 μM) at 37°C for 20 min. The fluorescence intensity of the HRCPs was observed by confocal laser scanning microscope (LEICA, Wetzlar, Germany). Detection conditions: excitation wavelength 504 nm, emission wavelength 529 nm, and the grating width of the excitation and emission wavelengths was 5 nm.

### The interaction between DNMT1 and PPARα promoter

The interaction between DNMT1 and PPARα promoter was examined by Chromatin Immunoprecipitation (ChIP) assay using Magna ChIP A/G Chromatin Immunoprecipitation Kit (Merck Millipore). In brief, HRCPs were fixed with 1% formaldehyde and glycine for 10 min to generate DNA–protein cross-links. The DNA–protein cross-links were degraded to chromatin fragments by sonication on ice for 3 min. The chromatin fragments were incubated with anti-PPARα (1:1000; Proteintech) or anti-IgG (1:2000; Proteintech) for immunoprecipitation. An aliquot of cell lysates served as input DNA control. The precipitated DNA was quantified by performing qRT-PCR. The primer sequences (5′–3′) of PPARα were shown as follows: forward: AGAGTAGTAGAGTTGGGTTTATTGA; reverse: AAAACAAAACTAAATTCAAAACAAA.

### Histological examination

Retinal tissues were separated from mice, and then fixed with 4% paraformaldehyde, and embedded in paraffin. After deparaffin and rehydration, 4 μm paraffin sections were obtained for histological examination. Subsequently, paraffin sections were stained using hematoxylin–eosin (HE) staining kit (Solarbio) to examine the damage of retinal tissues following the instruction of the manufacturer.

### Statistical analysis

Each assay was performed for three times. All data reported as mean ± standard deviation. SPSS 22.0 statistical software (IBM, Armonk, NY, USA) was used for statistical analysis. Two-tailed Student’s *t* test and one-way ANOVA were used to analyze the statistical difference. *P* < 0.05 was considered as a significant difference.

## Results

### HG promoted PPARα methylation and apoptosis of HRCPs.

**Fig. 1:**
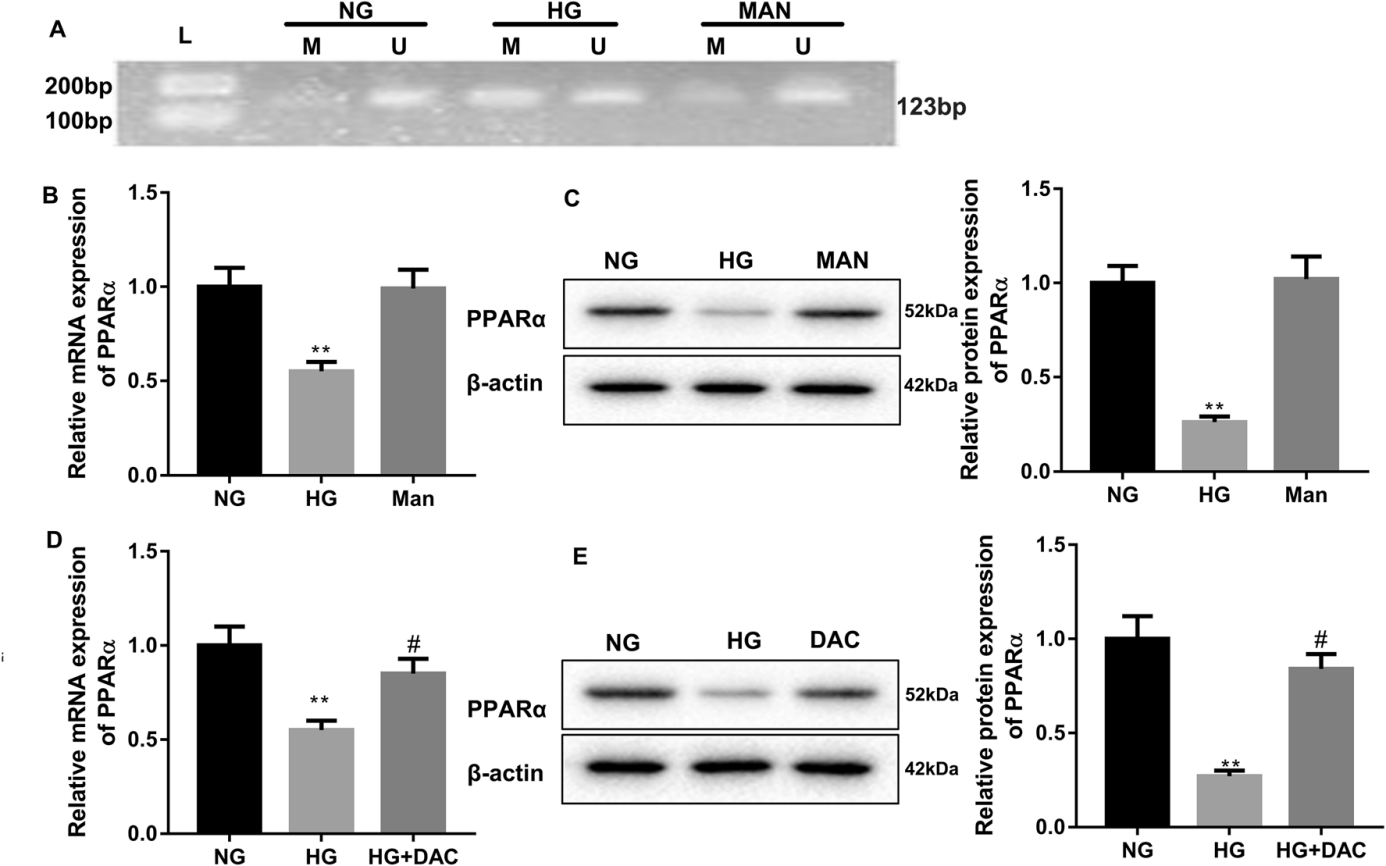
The methylation levels of PPARα in HRCPs were enhanced by HG treatment. HRCPs were treated with 5 mM glucose, 30 mM glucose or 5 mM glucose combined with 25 mM mannose. **A** The methylation levels of PPARα in HRCPs were detected by MSP. QRT-PCR (**B**) and WB (**C**) were performed to assess PPARα gene and protein expression in HRCPs. HRCPs were treated with 30 mM glucose combined with 10 mM DAC. QRT-PCR (**D**) and WB (**E**) were performed to examine the gene and protein expression of PPARα in HRCPs. *L* marker, *M* methylation, *U* unmethylation. **P < 0.01 vs. NG; ^#^*P* < 0.05 vs. HG

**Fig. 2:**
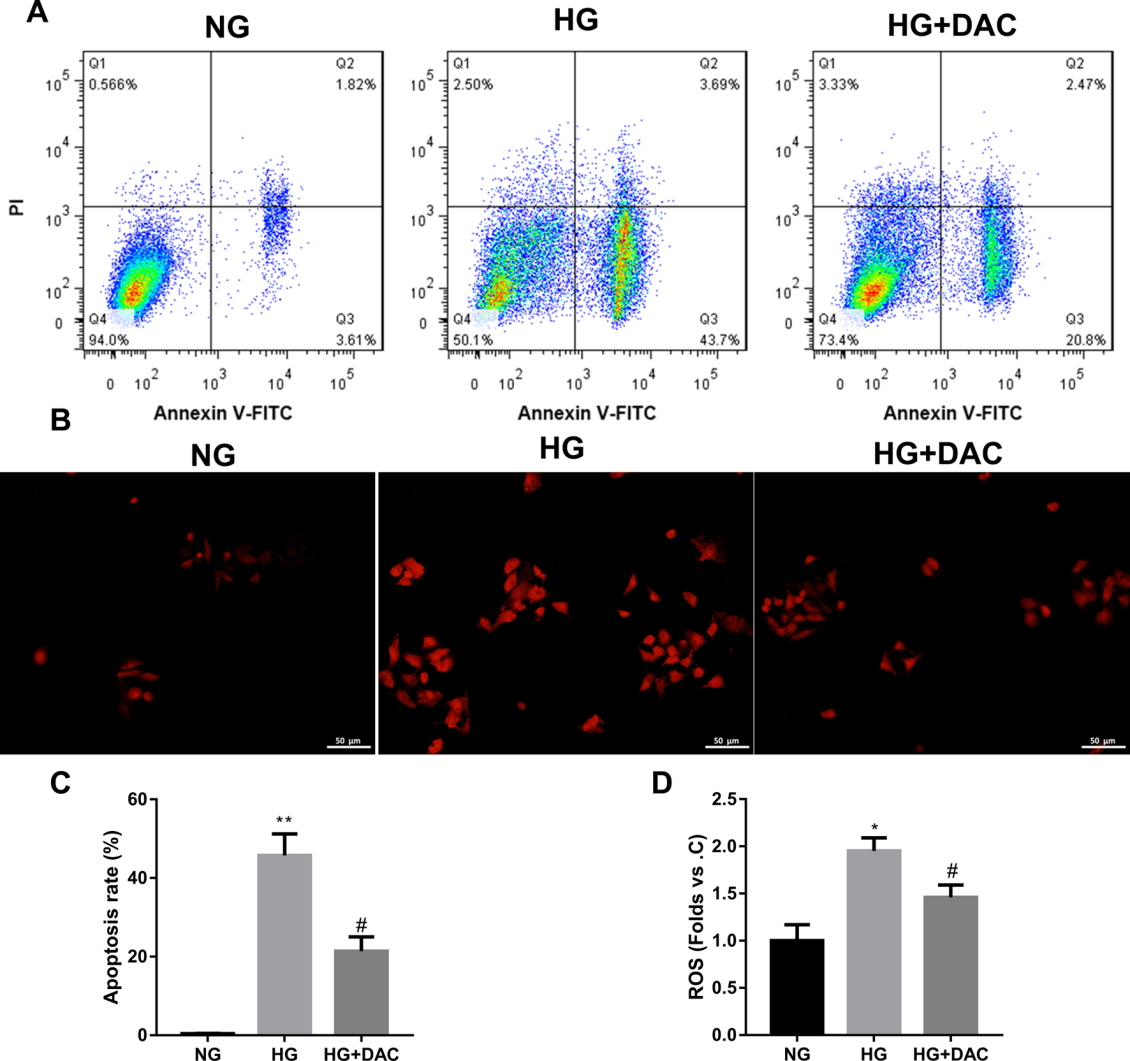
DAC treatment reduced apoptosis and the leves of ROS in HG-treated HRCPs. HRCPs were treated with 30 mM glucose combined with 10 mM DAC. **A**, **C** Flow cytometry was performed to estimate apoptosis of HRCPs. **B**, **D** The levels of ROS in HRCPs was detected by DCFH-DA fluorescent probe. *P < 0.05, **P < 0.01 vs. NG; ^#^*P* < 0.05 vs. HG

Previously, we have initially confirmed the role of PPARα in DR development. Whether PPARα methylation is associated with DR remains unknown. Here, we further investigated the biological role of PPARα methylation in DR. We first examined the methylation levels of PPARα in HG-treated HRCPs by MSP assay, showing that the methylation levels of PPARα were enhanced in HRCPs in the presence of HG (Fig. 1A). Next, we assessed the gene and protein expression of PPARα in HRCPs by qRT-PCR and WB. HG treatment notably repressed the gene and protein expression of PPARα in HRCPs as compared with NG group (Fig. 1B, C). There was no significant difference in the methylation and expression of PPARα between NG and Mannose groups (Fig. 1B, C). Subsequently, HG-treated HRCPs were incubated with DAC to inhibit DNA methyltransferase. QRT-PCR and WB data revealed that HG treatment caused a down-regulation of PPARα in HRCPs, which was partly rescued by DAC treatment (Fig. 1D, E). Moreover, flow cytometry was performed to estimate apoptosis of HRCPs. HG treatment aggravated apoptosis of HRCPs with respect to NG group. Apoptosis of HG-treated HRCPs was decreased in the presence of DAC (Fig. 2A, C). Furthermore, we estimated the levels of ROS in HRCPs by DCFH-DA fluorescent probe, showing that HG treatment led to a boost in the levels of ROS in HRCPs, which was partly abolished by DAC treatment (Fig. 2B, D). Thus, these findings showed that HG enhanced PPARα methylation and apoptosis of HRCPs.

### DNMT1 knockdown promoted PPARα expression and repressed HG-induced apoptosis in HRCPs

**Fig. 3:**
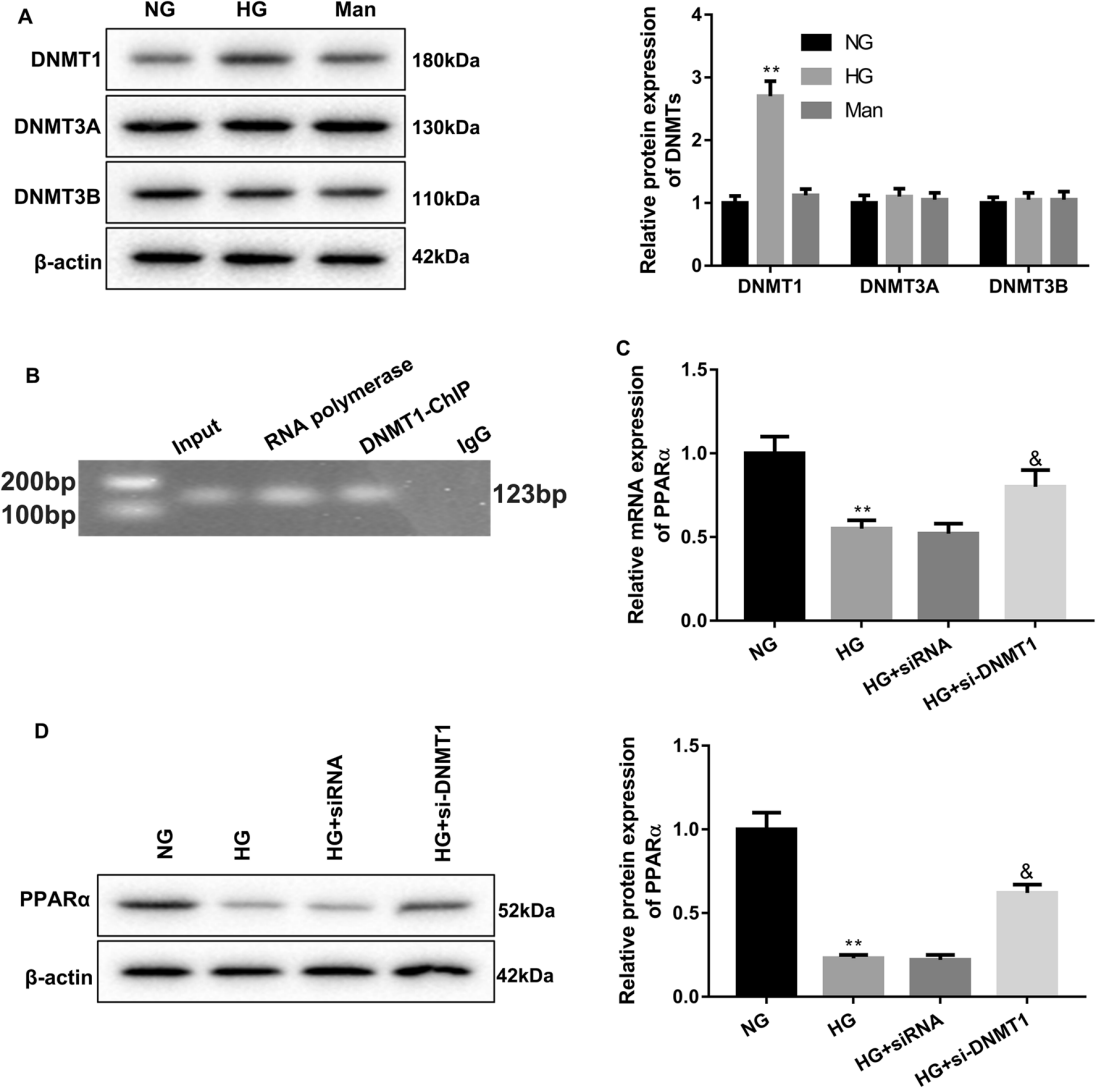
DNMT1 knockdown promoted PPARα expression in HG-treated HRCPs. HRCPs were treated with 5 mM glucose, 30 mM glucose or 5 mM glucose combined with 25 mM mannose. **A** WB was performed to assess the expression of DNMT1, DNMT3A and DNMT3B in HRCPs. **B** The interaction between DNMT1 and PPARα promoter was examined by ChIP assay. HRCPs were transfected with si-DNMT1 or siRNA, and then treated with 30 mM glucose. QRT-PCR (C) and WB (D) were performed to assess PPARα gene and protein expression in HRCPs. **P < 0.01 vs. NG; ^&^*P* < 0.05 vs. HG + siRNA

**Fig. 4:**
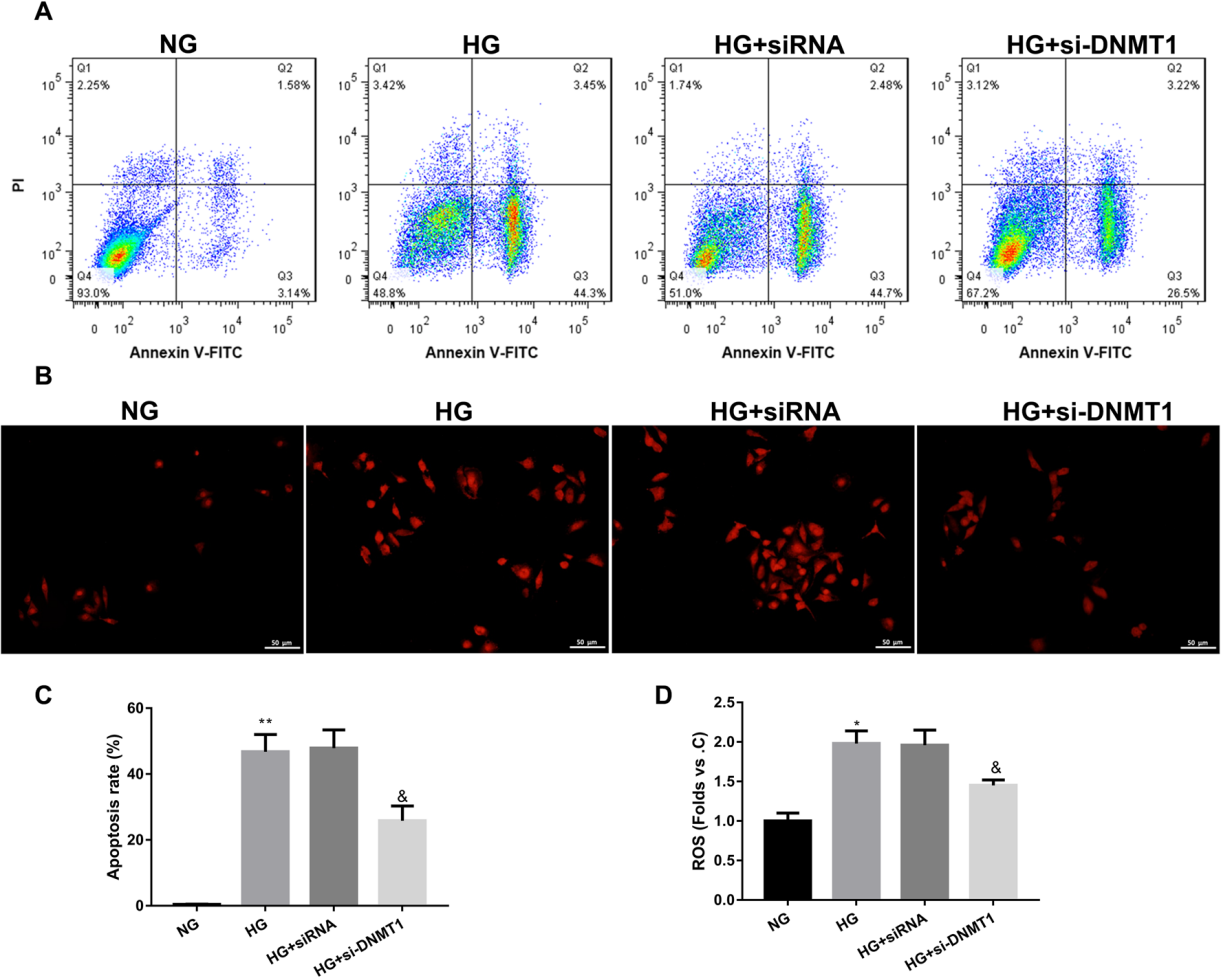
DNMT1 knockdown repressed apoptosis and the levels of ROS in HG-treated HRCPs. HRCPs were transfected with si-DNMT1 or siRNA, and then treated with 30 mM glucose. **A**, **C** Flow cytometry was performed to estimate apoptosis of HRCPs. **B**, **D** The levels of ROS in HRCPs was detected by DCFH-DA fluorescent probe. *P < 0.05, **P < 0.01 vs. NG; ^&^*P* < 0.05 vs. HG + siRNA

To determine the mechanism of PPARα methylation in HG-treated HRCPs, we assessed which methyltransferase (DNMT1, DNMT3A, DNMT3B) participated in the methylation of PPARα in HG-treated HRCPs by WB analysis. Figure 3A showed that HG significantly enhanced the expression of DNMT1 in HRCPs as compared with NG group. However, HG had no effect on the expression of DNMT3A and DNMT3B in HRCPs (Fig. 3A). Subsequently, we verified the relationship between DNMT1 and PPARα promoter by ChIP assay, indicating that DNMT1 interacted with PPARα promoter (Fig. 3B). Moreover, we silenced DNMT1 in HRCPs by transfection of si-DNMT1 or si-DNMT1-2. WB data showed that DNMT1 protein was severely decreased in HRCPs in the presence of si-DNMT1-1 and si-DNMT1-2, especially si-DNMT1-2 (Additional file 1: Figure S1). Thus, si-DNMT1-2 was used to silence DNMT1 in HRCPs. The DNMT1-silenced HRCPs were treated with HG. We examined the influence of DNMT1 deficiency on PPARα expression in HRCPs. QRT-PCR and WB data showed that HG led to a down-regulation of PPARα in HRCPs, which was partly rescued by DNMT1 deficiency (Fig. 3C, D). In addition, we assessed the effect of DNMT1 knockdown on apoptosis and ROS levels in HRCPs. The results of flow cytometry and DCFH-DA fluorescent probe showed that apoptosis and the levels of ROS in HG-treated HRCPs were notably increased with respect to NG group. HG treatment-mediated increase of apoptosis and ROS levels in HRCPs was repressed by DNMT1 silencing (Fig. 4A–D). Thus, these data suggested that HG-induced PPARα methylation in HRCPs was regulated by DNMT1.

### PPARα overexpression repressed apoptosis of HRCPs, which was rescued by DNMT1 up-regulation

**Fig. 5:**
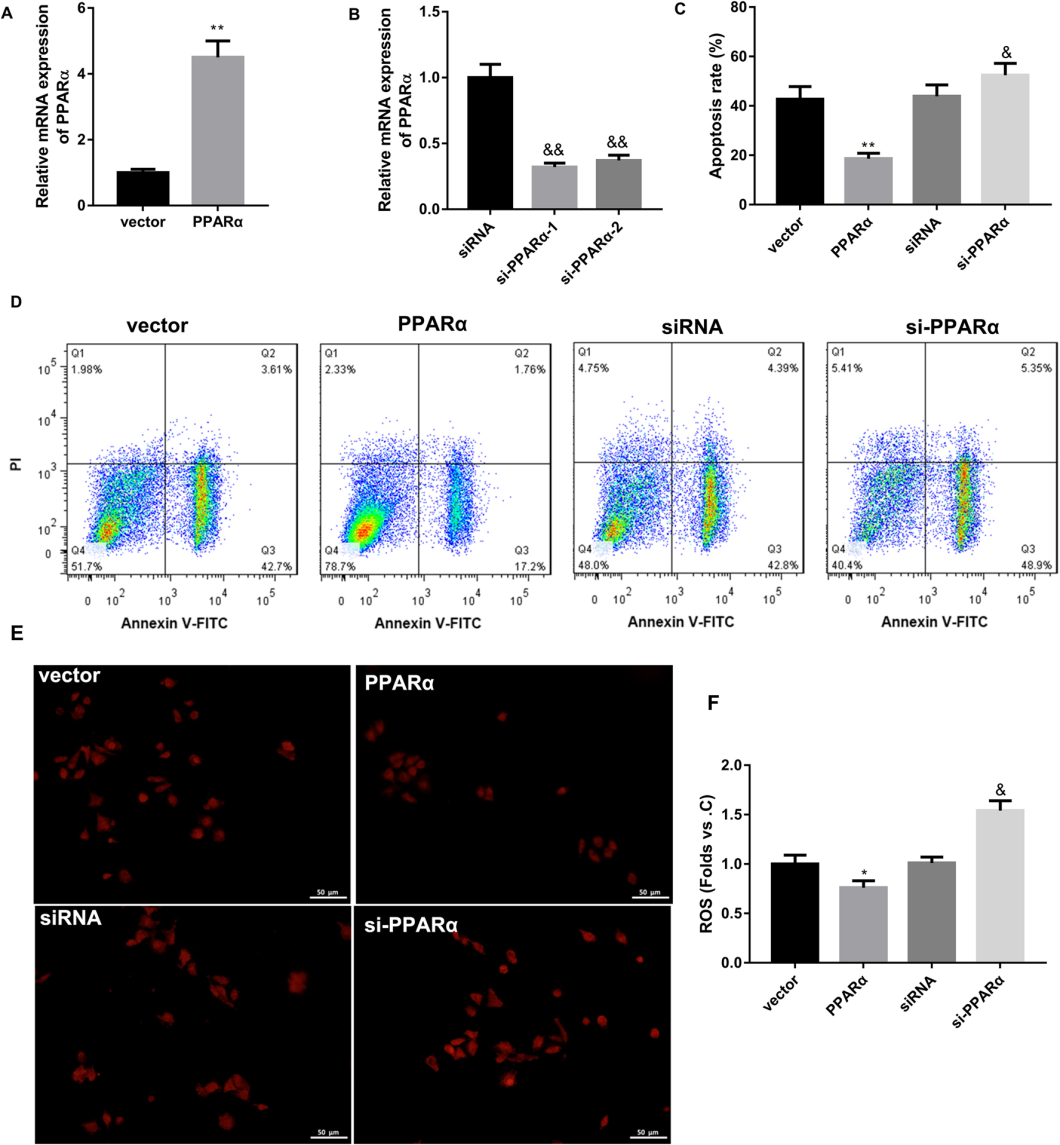
PPARα overexpression repressed apoptosis and ROS levels in HG-treated HRCPs. HRCPs were transfected with pCEP4-PPARα, pCEP4-NC, si-PPARα (si-PPARα-1 and si-PPARα-2) or siRNA. **A**, **B** QRT-PCR was performed to assess the expression of PPARα in HRCPs. The modified HRCPs were treated with 30 mM glucose. **C**, **D** Flow cytometry was performed to estimate apoptosis of HRCPs. **E**, **F** The levels of ROS in HRCPs was detected by DCFH-DA fluorescent probe. *P < 0.05, **P < 0.01 vs. Vector; ^&^*P* < 0.05, ^&&^*P* < 0.01 vs. siRNA

**Fig. 6:**
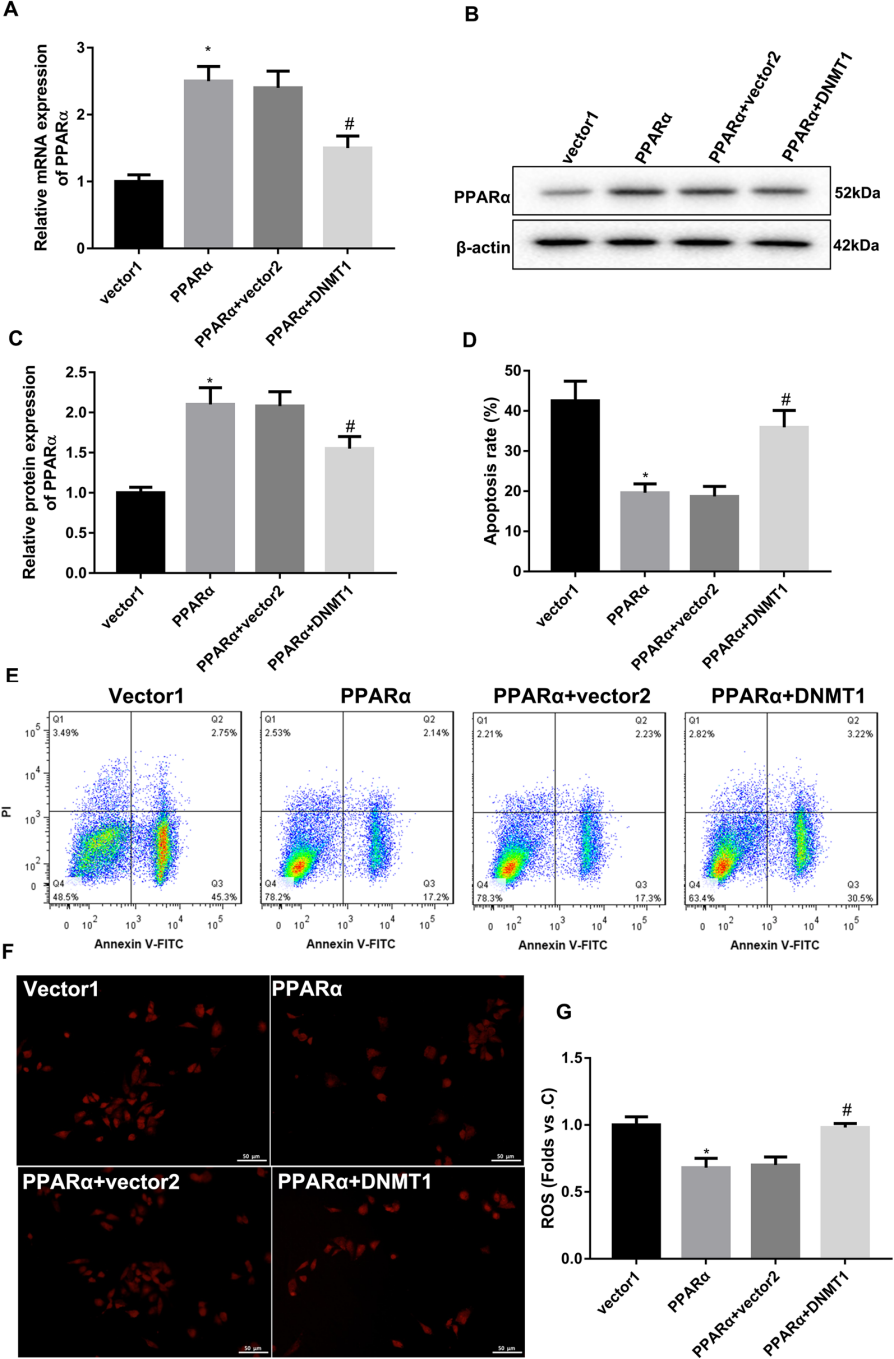
DNMT1 overexpression reversed the impact of PPARα overexpression on apoptosis and ROS levels in HG-treated HRCPs. HRCPs were co-transfected with pCEP4-PPARα, pCEP4-NC and pcDNA3.1-DNMT1 or pcDNA3.1-NC, and then treated with 30 mM glucose. QRT-PCR (**A**) and WB (**B**, **C**) were performed to assess PPARα gene and protein expression in HRCPs. **D**, **E** Flow cytometry was performed to estimate apoptosis of HRCPs. **F**, **G** The levels of ROS in HRCPs was detected by DCFH-DA fluorescent probe. *P < 0.05 vs. Vector1; ^#^*P* < 0.05 vs. PPARα + vector2

We further explored the biological role of DNMT1 in regulating PPARα expression and apoptosis in HRCPs. HRCPs were transfected with pCEP4-PPARα or si-PPARα for up-regulation or down-regulation of PPARα. The data of qRT-PCR revealed that the expression of PPARα was enhanced in HRCPs after transfected with pCEP4-PPARα (Fig. 5A). The expression of PPARα was severely decreased in HRCPs in the presence of si-PPARα-1 and si-PPARα-2, especially si-PPARα-1 (Fig. 5A, B). We then treated HRCPs with HG and estimated the effect of PPARα overexpression or down-regulation on apoptosis and ROS levels of the HRCPs. Figure 5C, D showed that PPARα up-regulation caused a decrease of apoptosis in HRCPs, whereas PPARα deficiency enhanced apoptosis of HRCPs. The levels of ROS in HRCPs was reduced by PPARα overexpression, and increased by PPARα silencing (Fig. 5E, F). In addition, HRCPs were co-transfected with pCEP4-PPARα and pcDNA3.1-DNMT1, and then treated with HG. The results obtained from qRT-PCR and WB revealed that PPARα overexpression enhanced the gene and protein expression of PPARα in HRCPs. The gene and protein expression of PPARα in HRCPs was severely down-regulated in the presence of pCEP4-PPARα and pcDNA3.1-DNMT1 (Fig. 6A–C). Furthermore, flow cytometry and DCFH-DA fluorescent probe were used to estimate apoptosis and the levels of ROS in HRCPs. PPARα up-regulation reduced apoptosis and the levels of ROS in HRCPs. The inhibiting effect of PPARα overexpression on apoptosis ROS levels in HRCPs was partly abolished by DNMT1 up-regulation (Fig. 6D–G). Taken together, these data confirmed that DNMT1 up-regulation abolished the inhibiting effect of PPARα overexpression on apoptosis of HRCPs.

### DAC treatment attenuated the damage of retinal tissues in DR mice by inhibiting the methylation levels of PPARα

**Fig. 7:**
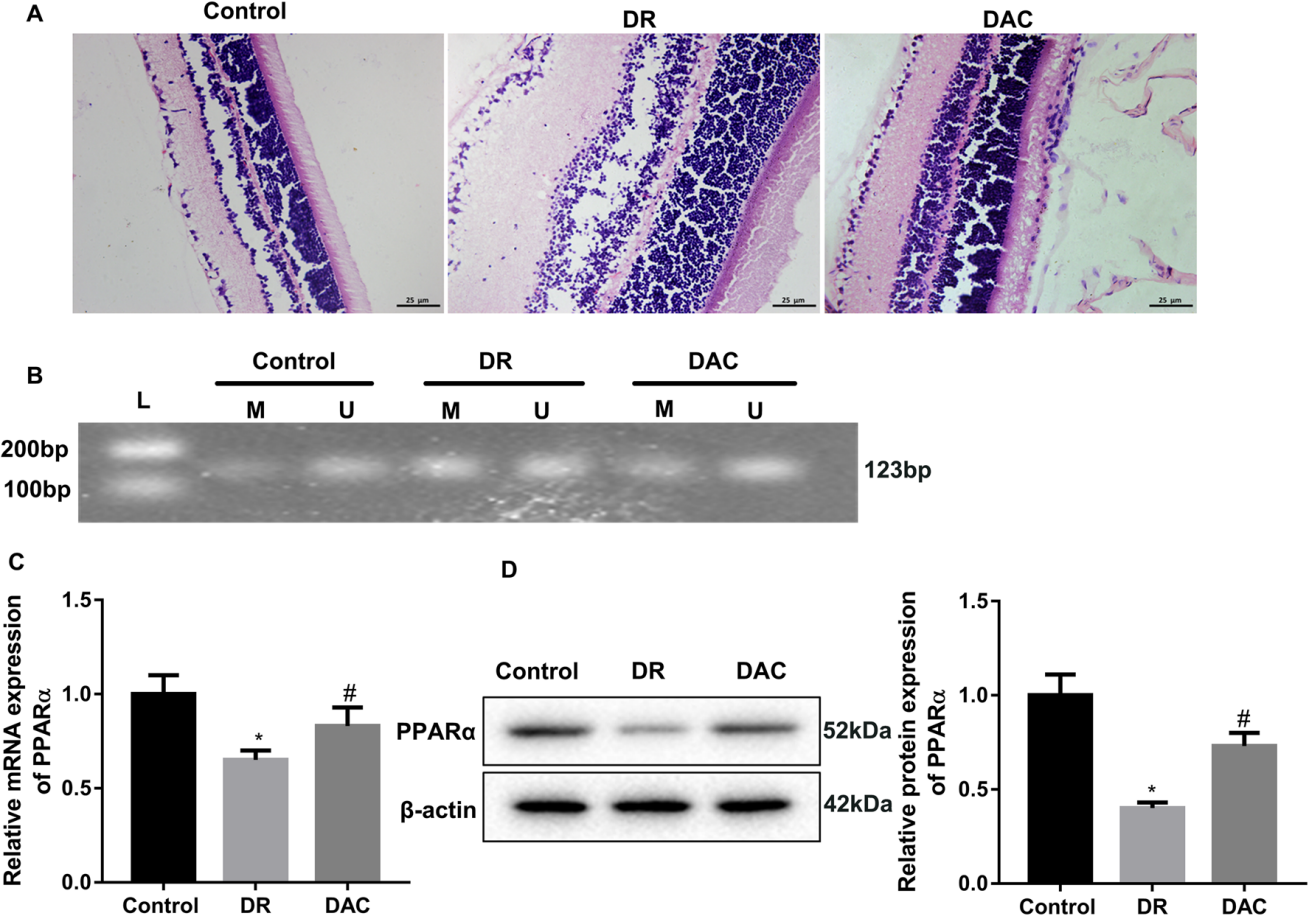
DAC treatment inhibited PPARα methylation and reduced damage of retinal tissues in DR mice. DR mouse model was established by intraperitoneal injection of STZ. DAC mice were received intraperitoneal injection of DAC. Normal mice served as control. **A** HE staining was performed to assess the pathological changes of retinal tissues. **B** The methylation levels of PPARα in retinal tissues were detected by MSP. QRT-PCR (**C**) and WB (**D**) were performed to assess the gene and protein expression of PPARα in retinal tissues. *L* marker, *M* methylation, *U* unmethylation.*P < 0.05 vs. Control; ^#^*P* < 0.05 vs. DR

We examined the effect of PPARα methylation on pathological changes of retinal tissues in DR mice. We constructed DR mouse model by intraperitoneal injection of STZ, and the DR mice were received DAC treatment to inhibit DNA methyltransferase. We observed the pathological changes of retinal tissues in mice by HE staining, showing that the control mice displayed a normal retinal tissue structure. The layers of retinal tissues were arranged regularly and clearly. Cell morphology was normal and compact. In STZ-induced DR mice, the layers of retinal tissues were loose and irregular. The number of ganglion cells was reduced, and the ganglion cell layer displayed obvious vacuolar degeneration. Cells in the inner and outer nuclear layers were arranged disorderly, and the cell density was reduced. However, the structure of each layer of retinal tissues was regular in STZ-induced DR mice following DAC treatment. Cells in the inner and outer nuclear layers were arranged neatly, and the cell density was increased in STZ-induced DR mice in the presence of DAC (Fig. 7A). Then, the methylation levels of PPARα in retinal tissues were detected by MSP assay. We found that the methylation levels of PPARα in DR mice were significantly enhanced with respect to control mice. Compared with DR mice, DAC treatment notably repressed the methylation levels of PPARα in mice (Fig. 7B). In addition, qRT-PCR and WB were performed to examine the effect of DAC treatment on PPARα expression in DR mice. PPARα mRNA and protein were notably down-regulated in retinal tissues of DR mice. However, the mRNA and protein expression of PPARα was increased in mice following treatment of DAC (Fig. 7C, D). Thus, these data demonstrated that DAC treatment attenuated the damage of retinal tissues in DR mice by inhibiting the methylation levels of PPARα.

## Discussion

In recent years, scholars have conducted in-depth researches on the mechanism of DNA methylation in the progression of DR. Maghbooli et al. have compared the levels of global DNA methylation between DR patients and diabetic patients without retinopathy, showing that DR patients display a higher global DNA methylation levels [31]. Thus, the levels of global DNA methylation may be a biomarker for DR. Previous study has confirmed that MnTBAP promotes the levels of 5mC and inhibits the interaction between MMP-9 promoter and DNMT1, thereby regulating the transcription of MMP-9 and repressing mitochondria damage in DR mice [32]. The transcription of MMP-9 in the retina is regulated by DNA methylation-hydroxymethylation process, thereby maintaining the homeostasis of mitochondria and inhibiting the occurrence and development of DR [33]. However, little is known about PPARα in DR. In the present work, we revealed the function of PPARα methylation in DR. HG treatment induced an increase of PPARα methylation levels and a down-regulation of PPARα in HRCPs. Moreover, HG treatment enhanced apoptosis and ROS levels in HRCPs. The influence conferred by HG treatment was effectively abolished by treatment of DNA methylation inhibitor, DAC. These data suggested that PPARα methylation was associated with HG-induced increase of apoptosis and ROS levels in HRCPs. Additionally, Qu et al. have confirmed the inhibiting effect of DAC on apoptosis of cloned embryos [34]. DAC represses apoptosis and oxidative stress of H_2_O_2_-treated SRA01/04 cells [35]. Thus, DAC may inhibit apoptosis and ROS levels in HRCPs.

PPARα methylation participates in the progression of various diseases. The steatotic hepatocyte models and non-alcoholic fatty liver disease rat models exhibit a down-regulation of PPAR-α and an increase of PPARα methylation, which are partly rescued by DAC or curcumin treatment [36]. High fructose promotes the methylation levels of PPARα and CPT1A in rat liver, suggesting that DNA methylation status of PPARα and CPT1A is closely associated with the pathogenesis of fructose-induced metabolic syndrome [37]. Betaine supplement alleviates hepatic triglyceride accumulation and improves antioxidant capacity in ApoE^−/−^ mice, which attributes to inhibit PPARα methylation and enhance PPARα expression [38]. Homocysteine-mediated DNA methylation of PPARα and PPARγ participates in the pathogenic mechanism of atherosclerosis [39]. In this work, we found that the expression of DNMT1 was enhanced in the HG-induced HRCPs, and DNMT1 interacted with PPARα promoter. We concluded that HG induced DNMT1-mediated PPARα methylation in HRCPs. Moreover, DNMT1 knockdown enhanced the expression of PPARα in HRCPs by reducing the methylation levels of PPARα. DNMT1 deficiency reduced HG-induced increase of apoptosis and ROS levels in HRCPs. Therefore, these results suggested that HG treatment enhanced apoptosis and ROS levels by promoting DNMT1-mediated PPARα methylation in HRCPs.

DNM methyltransferase is an important enzyme family in epigenetics that catalyzes and maintains DNA methylation. DNMT1 is a key enzyme for DNA to perform replication and repair, and maintain its normal methylation [40]. DNMT1 is highly expressed in hair follicle stem cells, and DNMT1-mediated miR-214-3p methylation promotes adipogenesis of hair follicle stem cells via MAPK1/p-ERK1/2 axis [41]. Long noncoding RNA PVT1 inhibits miR-18b-5p expression by recruiting DNMT1, and DNMT1 promotes miR-18b-5p methylation, thereby promoting gallbladder cancer proliferation [42]. These studies have confirmed the key role of DNMT1 in regulating DNA methylation of genes. However, whether PPARα methylation is regulated by DNMT1 has not been reported. Our data revealed that DNMT1 interacted with PPARα promoter, and repressed PPARα expression in HRCPs. Moreover, the inhibiting effect of PPARα overexpression on apoptosis and ROS levels in HRCPs was effectively rescued by DNMT1 overexpression. Thus, DNMT1 overexpression enhanced the methylation levels of PPARα and reduced PPARα expression, which contributed to elevate apoptotic cells and ROS levels in HRCPs. Finally, we constructed a DR mouse model by intraperitoneal injection of STZ, and verified the speculation by *in vivo* assays. The layers of retinal tissues in DR mice were loose and irregular. The number of ganglion cells was reduced, and the ganglion cell layer displayed obvious vacuolar degeneration. Cells in the inner and outer nuclear layers were arranged disorderly, and the cell density was reduced. Thus, the damage of the retinal tissues was manifested in the destruction of various layers of retina structure, which resulted in subsequent functional changes and vision loss. Many researches also have observed that the inner and outer nuclear layers are blurred, and the ganglion cell layer is destroyed in the retina of DR mice and rats [43–45]. A previous study has confirmed that there are changes in nerve fiber function before detectable microvascular changes appear in the fundus of diabetic patients, and retinal nerve function degeneration occurs in the early stage of DR [46]. The pathological changes of retinal tissues observed in our data also confirmed this view. Additionally, DAC treatment effectively reduced damage of retinal tissues in DR mice. DR mice exhibited a high methylation level of PPARα, and a down-regulation of PPARα, which was abrogated by DAC treatment-mediated inhibition of PPARα methylation. This, these data demonstrated that DNMT1-mediated PPARα methylation promoted apoptosis of HRCPs and aggravated damage of retinal tissues, thereby accelerating the progression of DR (Additional file 2: Figure S2).

Previous study has shown that DNMT1-mediated MEG3 methylation promotes endothelial-mesenchymal transition in DR, and thus facilitates DR development [21]. Biswas et al. have demonstrated that inhibition of MALAT1 methylation enhances the levels of inflammatory factors in human retinal endothelial cells, which contributes to accelerate DR progression [47]. Thus, DNA methylation has a complex function in the development of DR. The specific mechanism of DNA methylation in DR still needs further in-depth study. Additionally, inflammation and macrophage infiltration are closely associated with the development of DR [48, 49]. This work only initially explored the effect of DNMT1-mediated PPARα methylation on apoptosis and ROS in HRCPs. Whether PPARα methylation can affect inflammation and macrophage infiltration in DR is still unclear, which is a shortcoming of our research. We will conduct research on this issue in future work.

## Conclusions

In conclusion, our data demonstrate that DNMT1-mediated PPARα methylation promotes apoptosis of HRCPs and aggravates damage of retinal tissues in DR mice. Thus, this study may highlight novel insights into the pathogenic mechanisms of DR.

## Supplementary Information

**Additional file 1: Figure S1.** The protein expression of DNMT1 in HRCPs. WB was performed to assess the protein expression of DNMT1 in HRCPs following transfection of si-DNMT1-1, si-DNMT1-2 or siRNA.

**Additional file 2: Figure S2.** Schematic representation of the functional role of PPARα in DR.

## Data Availability

The datasets used and/or analysed during the current study are available from the corresponding author on reasonable request.

## Abbreviations

ChIP: Chromatin immunoprecipitation
DAC: 5-Aza-2-deoxycytidine
DNMT1: DNA methyltransferase-1
DR: Diabetic retinopathy
FBS: Fetal bovine serum
HE: Hematoxylin–eosin
HG: High glucose
HRCPs: Human retinal capillary pericytes
Man: Mannose
MSP: Methylation-specific PCR
NG: Normal glucose
PBS: Phosphate buffer saline
PPARs: Peroxisome proliferator-activated receptors
qRT-PCR: Quantitative real-time PCR
ROS: Reactive oxygen species
siRNA: Small interference RNA
STZ: Streptozotocin
WB: Western blot
