# Supplementary figures and images for: DNMT1-mediated PPARα methylation aggravates damage of retinal tissues in diabetic retinopathy mice
Source: Biol Res. 2021 Aug 6;54:25. doi: 10.1186/s40659-021-00347-1 (PMC8348846; doi:10.1186/s40659-021-00347-1)

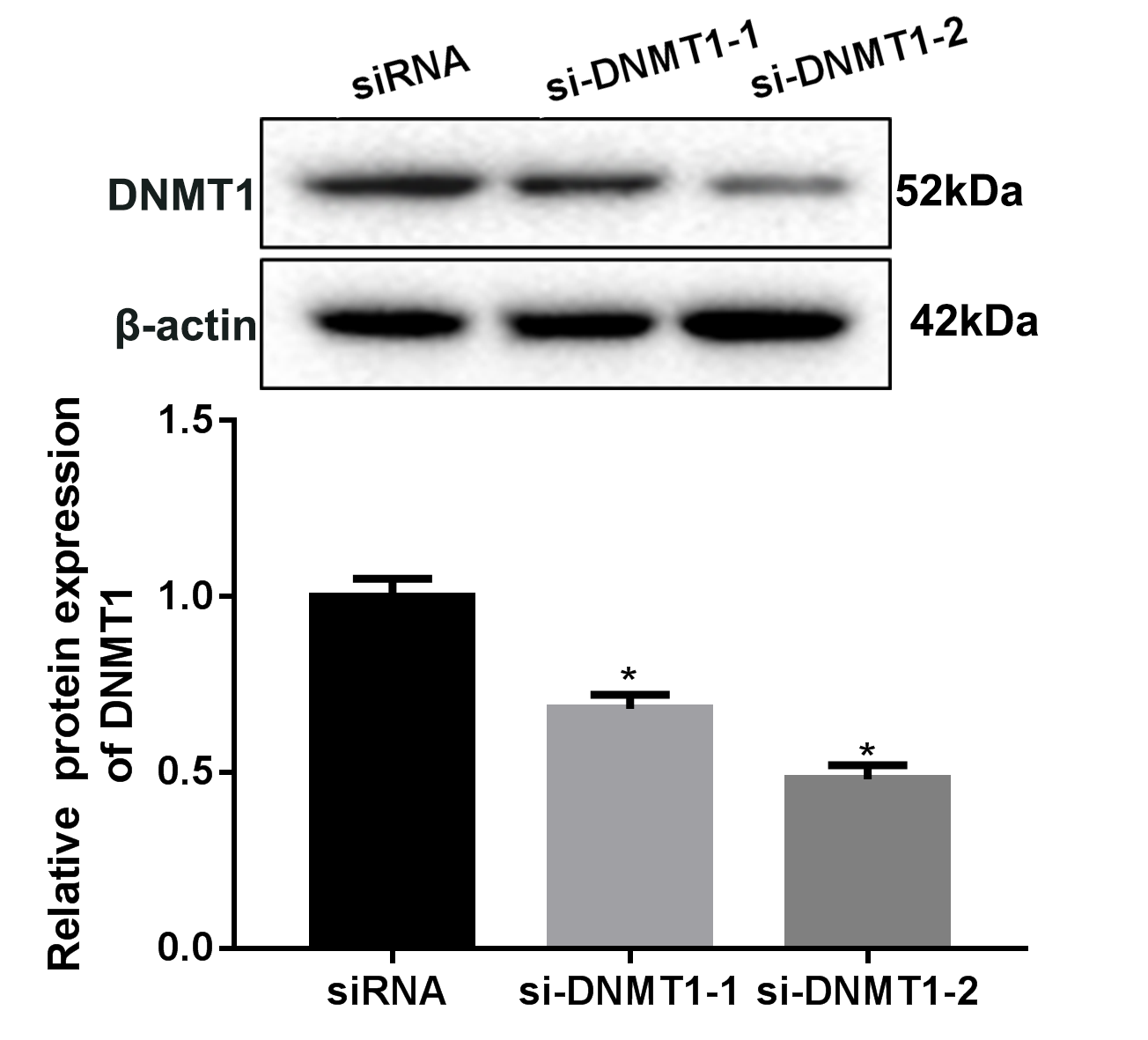

Supplement: Supplementary file 1 — Additional file 1: Figure S1. The protein expression of DNMT1 in HRCPs. WB was performed to assess the protein expression of DNMT1 in HRCPs following transfection of si-DNMT1-1, si-DNMT1-2 or siRNA. [file 40659_2021_347_MOESM1_ESM.tif]

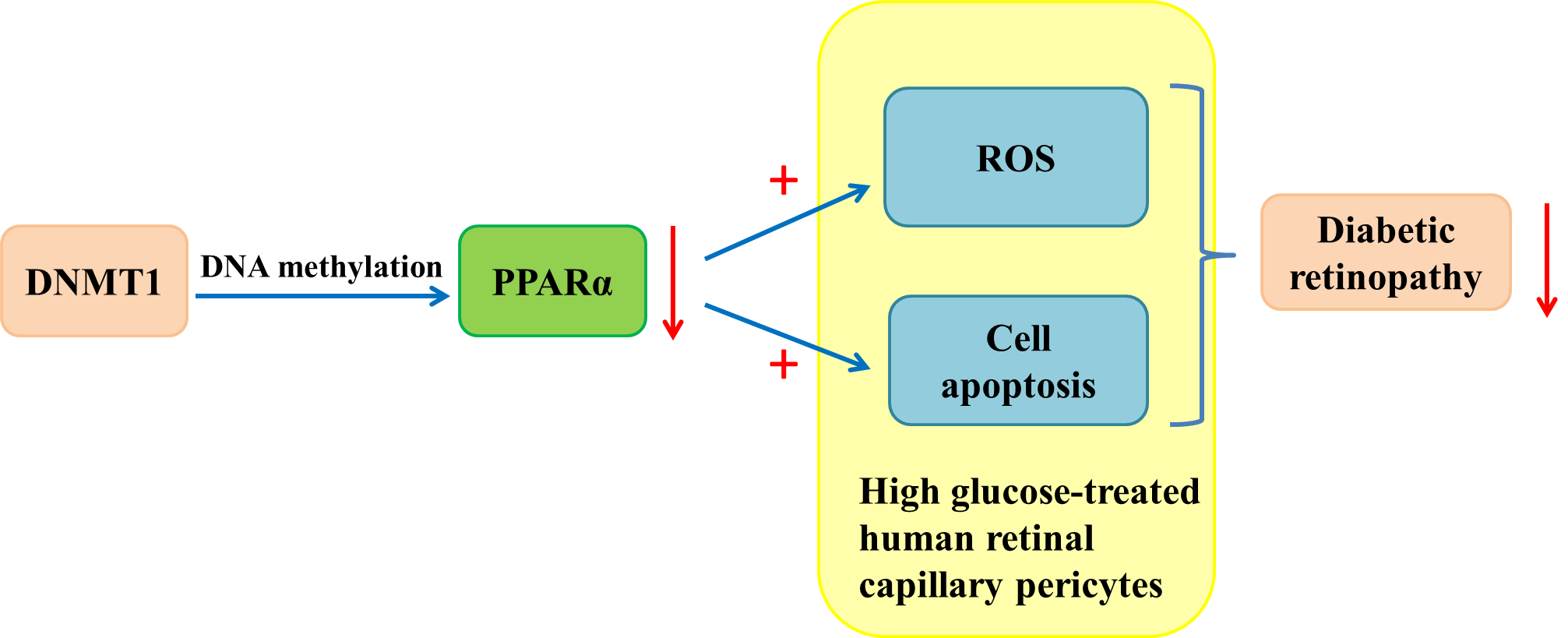

Supplement: Supplementary file 2 — Additional file 2: Figure S2. Schematic representation of the functional role of PPARα in DR. [file 40659_2021_347_MOESM2_ESM.tif]
